# Association of compliance with COVID-19 public health measures with depression

**DOI:** 10.1038/s41598-022-17110-5

**Published:** 2022-08-05

**Authors:** Ju An Byun, Tae Jun Sim, Tae Yoon Lim, Sung-In Jang, Seung Hoon Kim

**Affiliations:** 1 Premedical Courses, Yonsei University College of Medicine, Seoul, Republic of Korea; 2 Department of Preventive Medicine, Yonsei University College of Medicine, Seoul, Republic of Korea; 3 Institute of Health Services Research, Yonsei University, Seoul, Republic of Korea

**Keywords:** Psychiatric disorders, Public health, Infectious diseases

## Abstract

Although previous studies have demonstrated increased depression related to COVID-19, the reasons for this are not well-understood. We investigated the association of compliance with COVID-19 public health measures with depression. Data from the 2020 Korea Community Health Survey were analyzed. The main independent variable was compliance with rules based on three performance variables (social distancing, wearing a mask in indoor facilities, and outdoors). Depression was assessed using Patient Health Questionnaire-9 scores. Of 195,243 participants, 5,101 participants had depression. Bad and moderate performance scores for compliance were associated with depression (Bad score, men: adjusted odds ratio [aOR] = 2.24, 95% confidence interval [CI] = 1.29–3.87; women: aOR = 2.42, 95% CI = 1.42–4.13; moderate score, men: aOR = 1.31, 95% CI = 1.02–1.68; women: aOR = 1.28, 95% CI = 1.07–1.53). In the subgroup analysis, among the quarantine rules, not wearing a mask indoors was the most prominently associated with depression. In participants with a high level of education, non-compliance with quarantine rules was significantly associated with depression. People who do not comply with public health measures are more likely to be depressed. The preparation and observance of scientific quarantine rules can help mental health in the ongoing COVID-19 pandemic and another infectious disease pandemic that may come.

## Introduction

The coronavirus disease 2019 (COVID-19) outbreak, which started in 2019, has completely changed the lives of people around the world. According to the World Health Organization (WHO), as of June 2022, more than 500 million cases have occurred worldwide, and about 6.3 million people have died from COVID-19^1^. While many governments seek treatments and vaccines, most governments around the world have implemented various forms of anti-epidemic policies to prevent the further spread of COVID-19^2,3^. At the individual level, measures such as wearing a mask, measuring body temperature when entering a building, and sanitizing hands were implemented. Group-level controls were also implemented to restrict face-to-face interactions, such as recommending physical or social distancing and forcing employees to work from home^4–6^.

The outbreak of COVID-19 also has caused a variety of psychological problems such as panic disorder, anxiety, and depression that can occur after major economic crises or natural disasters^7–10^. In patients infected with COVID-19, anxiety symptoms and fears regarding uncertainty about treatment and health outcomes can affect their mental health^11^. A recent study of survivors of COVID-19 infection reported that markers related to the immune response were associated with anxiety and depression^12^, and the frequency of depressive symptoms ≥ 12 weeks after COVID-19 infection was reported to be 11–28%^13^. Furthermore, health care providers who have had direct contact with COVID-19 patients are more likely to experience anxiety and depression^14^.

The burden of mental health problems for the general population during COVID-19 continues to be reported, even when not under special circumstances, such as those infected with COVID-19 or the medical staff treating them^15^. Adverse mental health outcomes may arise from physical symptoms resembling COVID-19 infection mediated by the perceived impact of the pandemic and the absence of health information^16^. Moreover, public health interventions implemented in several countries, such as lockdown and quarantine measures, may have affected mental health, including causing anxiety and depression, during COVID-19 pandemic^5,17^. According to a previous study, mental health was affected by the strictness of quarantine policies and the number of deaths caused by COVID-19 in the Netherlands, UK, and France^18^.

The lack of interaction between people and restrictions on freedom may have significant impacts on the enjoyment of life as a human being^19^. Negative effects on the economic well-being and quality of life have been reported after national social distancing measures due to COVID-19, suggesting that public health interventions to prevent the spread of infection are affecting the lives of the general population as a whole^20^. Furthermore, these special circumstances, including isolation and social distancing, are likely to contribute to the frustration, boredom, and depressed mood of the general population^19,21^.

With regard to the mental well-being of the general population, a rapid public health response may be more helpful than a late public health response^22^. Moreover, adherence to social distancing and perceived effectiveness of social distancing are associated with lower levels of anxiety and depressive symptoms^23^. In addition, in Poland, where the use of a mask was not recommended in the early stages of COVID-19 pandemic, it was found that the level of depression and stress was higher compared to that in China, where masks were recommended^24^. These findings imply that, even if individual freedom is restricted, there is a positive effect on mental health if people perceive that implementing and observing quarantine rules is for the well-being of the community and their own health.

Taken together, not only isolation measures but also public health measures, such as social distancing and wearing masks, that restrict individual freedom can affect the mental health of the general population who have experienced the outbreak of COVID-19. However, considering that humans are beings with free will, it should be considered that the government's establishment of quarantine rules and the people's observance of them are different dimensions when considering public health measures in a pandemic situation.

Thus, this study evaluated the association of compliance with COVID-19 public health measures with depression in Korean adults. Furthermore, this study aimed to serve as a basis for preparing an efficient and effective response plan for public health policies, not only for the current crisis but also for other infectious diseases to come in the future, by estimating the impact of public health policies on mental health.

## Methods

### Study population and data

This study used data from the 2020 Community Health Survey (CHS) in South Korea. The CHS is a nationwide population-based survey, the purpose of which is to obtain the health data of South Korean citizens who are adults ≥ 19 years. This health data includes information about mental health, medical service usage, and diet. In particular, the 2020 CHS, conducted from August 16 to October 31, 2020, included data related to COVID-19, such as the practicing of social distancing. The Korea Disease Control and Prevention Agency conducts the CHS every year by visiting and interviewing selected family households. In CHS, stratified cluster sampling method and systematic sampling method were used to select sample area and sample household, respectively^25^. The dataset used in this study can be provided through a predetermined procedure after entering certain information on the CHS’s official website (https://chs.kdca.go.kr/chs/).

A total of 229,269 participants were involved in the 2020 CHS. In this study, participants whose answers were “Refused to respond,” “Don’t know,” or “Unmatched” in the survey (n = 34,026) were excluded. As a result, 195,243 participants (89,135 men and 106,108 women) were selected for this study. Since the CHS is a survey conducted by the government for public welfare, ethics approval for the CHS was waived by the Bioethics and Safety Act, 2015. This study adhered to the tenets of the Declaration of Helsinki and all methods were performed in accordance with the relevant guidelines and regulations.

### Depression

Depression was the primary outcome of this study. The PHQ-9 is an instrument for screening, diagnosing, monitoring, and measuring the severity of depression^26,27^. The Korean version of PHQ-9 has been verified for validity and reliability in a population-based survey^28^. The PHQ-9 consists of 9 items measuring the frequency of depressive symptoms over the past two weeks, and each item is scored on a scale of 0–3. The sum of the scores ranges from 0–27, with higher scores indicating more severe depression. According to the definition of depression on the PHQ-9 test, participants with scores ≥ 10 in the PHQ-9 test were defined as having depression^29^. Participants with scores < 10 were defined as normal.

### Compliance with COVID-19 public health measures

The main independent variable was compliance with COVID-19 public health measures, which was determined based on responses to questions regarding social distancing and wearing masks. Social distancing was evaluated by one question, “Do you practice social distancing by maintaining distance?” The answers to the question were either “Absolutely,” “Yes,” or “No.” Participants who answered “Absolutely” or “Yes” to the question were given one point, and those who answered “No” were given zero. The wearing of masks was evaluated based on two questions, “Do you wear a mask in indoor facilities?” and “Do you wear a mask outdoor when social distancing is difficult?” The possible answers to these two questions were “Absolutely,” “Yes,” or “No.” Those who answered “Absolutely” or “Yes” to the question regarding wearing a mask in indoor facilities were given one point and those who answered “No” were given zero. Those who answered “Absolutely” or “Yes” to the question regarding wearing a mask outdoors were given one point and others were given zero.

Based on these questions, the participants were given a COVID-19 compliance performance score. For each participant, the performance score was the sum of the points for the three questions above, hence the larger the score the better the compliance. Finally, we classified a performance score of 0 to 1 as bad, a score of 2 as moderate, and a score of 3 as good.

### Covariates

The covariates for this study included age (19–29, 30–39, 40–49, 50–59, 60–69, or ≥ 70 years), education level (did not graduate high school or graduated high school), employment status (white, pink, or blue collar or none), household income (low, middle low, middle high, or high), smoking status (yes or no), alcohol consumptions (once/month and more than or less than once/month), physical activity (high or low, with high indicating walked ≥ 30 min/day for ≥ 5 d/week), chronic disease history (hypertension and diabetes), and subjective health status (good, normal or bad). Subjective health status was categorized based on the response to the question, “How do you think of your own health status?”.

### Statistical analysis

All analyses were conducted separately by sex to account for sex-specific differences in rates of depression^30^. To assess the differences between groups of participants with depression and groups of those without depression for each sex, chi-squared tests were performed for categorical variables. After adjusting for covariates, multiple logistic regression analysis was used to evaluate the association of compliance with COVID-19 public health measures, using the performance score, with depression for men and women. Odds ratios (ORs) and 95% confidence intervals (CIs) were calculated. The association between the performance score for each question and depression in men and women was also evaluated. Finally, the association of the performance score with depression for each sex stratified by education level was evaluated. All analyses were performed using Statistical Analysis Software (SAS, version 9.4, SAS, Inc., Cary, NC, USA). To account for the complex and stratified sampling design, a weighted logistic regression procedure was used^31^. The *p* values were two-sided, and statistical significance was assumed when *p* < 0.05.

### Ethics approval

Since the CHS is a survey conducted by the government for public welfare, ethics approval for the CHS was waived by the Bioethics and Safety Act, 2015.

## Results

Table 1 presents the general characteristics of male and female participants along with the performance scores. Among the 195,243 participants, the number of participants who had good COVID-19 quarantine rules performance score was 184,746 (94.62%), moderate score was 9,249 (4.74%), and bad score was 1,248 (0.64%). For each of these categories, the number of participants who showed depression based on the PHQ-9 scores was 4,684 (2.54%), 354 (3.83%), and 63 (5.05%), respectively (percentages reflect the number in each category). Among the 5,101 participants who showed depression, the number of men was 1,620 and that of women was 3,481.

**Table 1:** General characteristics of study subjects.

Variables	Depression (PHQ-9 ≥ 10)									
	Men					Women				
	Total		Yes		*p*-value	Total		Yes		*p*-value
	N	%	N	%		N	%	N	%	
Total (n = 195,243)	89,135	100	1,620	1.82		106,108	100	3,481	3.28	
**COVID-19 quarantine rules performance score**					< 0.001					< 0.001
Bad (0–1 point)	713	0.8	30	4.2		535	0.5	33	6.2	
Moderate (2 points)	4,591	5.2	135	2.9		4,658	4.4	219	4.7	
Good (3 points)	83,831	94.0	1,455	1.7		100,915	95.1	3,229	3.2	
**Age (years)**					< 0.001					< 0.001
19–29	11,463	12.9	245	2.1		12,247	11.5	537	4.4	
30–39	10,968	12.3	244	2.2		11,838	11.2	430	3.6	
40–49	15,176	17.0	250	1.6		16,982	16.0	438	2.6	
50–59	17,837	20.0	247	1.4		21,064	19.9	537	2.5	
60–69	17,165	19.3	247	1.4		20,702	19.5	519	2.5	
70–	16,526	18.5	387	2.3		23,275	21.9	1,020	4.4	
**Educational level**					< 0.001					< 0.001
Under high school	21,506	24.1	570	2.7		40,475	38.1	1,563	3.9	
Graduated high school	67,629	75.9	1,050	1.6		65,633	61.9	1,918	2.9	
**Employment status**					< 0.001					< 0.001
White collar	19,880	22.3	250	1.3		19,356	18.2	475	2.5	
Pink collar	9,397	10.5	173	1.8		16,558	15.6	468	2.8	
Blue collar	35,754	40.1	423	1.2		20,864	19.7	432	2.1	
None or else	24,104	27.0	774	3.2		49,330	46.5	2,106	4.3	
**Household income**					< 0.001					< 0.001
Low	10,013	11.2	447	4.5		17,865	16.8	987	5.5	
Middle low	27,834	31.2	541	1.9		32,953	31.1	1,154	3.5	
Middle high	24,060	27.0	350	1.5		25,388	23.9	696	2.7	
High	27,228	30.5	282	1.0		29,902	28.2	644	2.2	
**Smoking status**					< 0.001					< 0.001
No	60,024	67.3	934	1.6		103,357	97.4	3,154	3.1	
Yes	29,111	32.7	686	2.4		2,751	2.6	327	11.9	
**Alcohol consumption**					< 0.001					< 0.001
< 1time/month	33,635	37.7	742	2.2		71,093	67.0	2,324	3.3	
≥ 1 times/month	55,500	62.3	878	1.6		35,015	33.0	1,157	3.3	
**Physical activity**					< 0.001					< 0.001
Low	50,540	56.7	1,061	2.1		66,284	62.5	2,510	3.8	
High	38,595	43.3	559	1.4		39,824	37.5	971	2.4	
**Chronic disease**^a^					< 0.001					< 0.001
None	60,799	68.2	987	1.6		74,026	69.8	2,213	3.0	
Has disease	28,336	31.8	633	2.2		32,082	30.2	1,268	4.0	
**Subjective health status**					< 0.001					< 0.001
Good	48,765	54.7	313	0.6		47,638	44.9	586	1.2	
Normal	32,063	36.0	581	1.8		43,120	40.6	1,220	2.8	
Bad	8,307	9.3	726	8.7		15,350	14.5	1,675	10.9	

*PHQ-9* Patient Health Questionnaire-9.

^a^A chronic disease was defined as a diagnosis of hypertension or diabetes mellitus; The number of chronic diseases is the sum of the number of the above diagnoses.

Table 2 shows the factors associated with depression. After adjusting for all covariates, those who showed bad performance scores were more likely to have depression than those who showed good performance scores. Using good performance score as the reference, the aORs for men were as follows: moderate, aOR = 1.31, 95% CI: 1.02–1.68; bad, aOR = 2.24, 95% CI: 1.29–3.87. Similarly, the ORs for women were as follows: moderate, aOR = 1.28, 95% CI: 1.07–1.53; bad, aOR = 2.42, 95% CI: 1.42–4.13.

**Table 2:** Factors associated with depression (PHQ − 9 ≥ 10).

Variables	Depression (PHQ-9 ≥ 10)					
	Men			Women		
	aOR	95% CI	*p*-value	aOR	95% CI	*p*-value
**Covid-19 quarantine rules performance score**						
Bad (0–1 point)	2.24	(1.29–3.87)	0.004	2.42	(1.42–4.13)	0.001
Moderate (2 points)	1.31	(1.02–1.68)	0.034	1.28	(1.07–1.53)	0.008
Good (3 points)	1.00			1.00		
**Age (years)**						
19–29	4.48	(3.42–5.85)	< 0.001	3.51	(2.84–4.34)	< 0.001
30–39	4.72	(3.61–6.15)	< 0.001	2.82	(2.27–3.49)	< 0.001
40–49	2.33	(1.79–3.02)	< 0.001	1.87	(1.51–2.31)	< 0.001
50–59	1.82	(1.42–2.33)	< 0.001	1.56	(1.31–1.87)	< 0.001
60–69	1.17	(0.94–1.46)	0.162	1.00	(0.86–1.17)	0.961
70–	1.00			1.00		
**Educational level**						
Under high school	1.49	(1.23–1.80)	< 0.001	1.14	(0.98–1.32)	0.093
Graduated high school	1.00			1.00		
**Employment status**						
White collar	0.82	(0.67–1.01)	0.057	0.84	(0.74–0.97)	0.014
Pink collar	0.89	(0.71–1.11)	0.294	1.05	(0.91–1.20)	0.517
Blue collar	0.59	(0.50–0.71)	< 0.001	0.67	(0.58–0.78)	< 0.001
None or else	1.00			1.00		
**Household income**						
Low	3.06	(2.43–3.84)	< 0.001	2.31	(1.98–2.71)	< 0.001
Middle low	1.78	(1.48–2.14)	< 0.001	1.66	(1.46–1.89)	< 0.001
Middle high	1.41	(1.17–1.69)	0.001	1.33	(1.16–1.52)	< 0.001
High	1.00			1.00		
**Smoking status**						
No	0.63	(0.55–0.73)	< 0.001	0.36	(0.31–0.42)	< 0.001
Yes	1.00			1.00		
**Alcohol consumption**						
< 1time / month	1.08	(0.94–1.24)	0.276	0.77	(0.70–0.85)	< 0.001
≥ 1 times / month	1.00			1.00		
**Physical activity**						
Low	1.15	(1.01–1.31)	0.031	1.34	(1.22–1.48)	< 0.001
High	1.00			1.00		
**Chronic disease**^a^						
None	0.97	(0.83–1.13)	0.696	1.03	(0.92–1.15)	0.653
Has disease	1.00			1.00		
**Subjective health status**						
Good	0.06	(0.05–0.08)	< 0.001	0.08	(0.07–0.09)	< 0.001
Normal	0.20	(0.17–0.23)	< 0.001	0.21	(0.19–0.24)	< 0.001
Bad	1.00			1.00		

*PHQ-9* Patient Health Questionnaire-9; *aOR* adjusted odds ratio; *CI* confidence interval.

^a^A chronic disease was defined as a diagnosis of hypertension or diabetes mellitus; The number of chronic diseases is the sum of the number of the above diagnoses.

Table 3 shows the association of social distancing and wearing mask with depression. Participants who did not practice social distancing in both men and women were more likely to show depression (Men: aOR = 1.31, 95% CI: 1.02–1.68; Women: aOR = 1.38, 95% CI: 1.15–1.66). Men and women who answered that they did not wear masks at indoor facilities had a higher risk of depression than those who answered that they wore masks (Men: aOR = 2.32, 95% CI: 1.33–4.03, Women: aOR = 1.85, 95% CI: 1.07–3.18). Among men, not wearing a mask when social distancing was difficult was significantly associated with depression (aOR = 1.82, 95% CI: 1.14–2.91).

**Table 3:** Association of social distancing and wearing mask with depression.

Variables	Depression (PHQ-9 ≥ 10)					
	Men			Women		
	aOR	95% CI	*p*-value	aOR	95% CI	*p*-value
**Social distancing**						
Bad (0 point)	1.31	(1.02–1.68)	0.035	1.38	(1.15–1.66)	< 0.001
Good (1 point)	1.00			1.00		
**Wearing mask in indoor facilities**						
Bad (0 point)	2.32	(1.33–4.03)	0.003	1.85	(1.07–3.18)	0.027
Good (1 point)	1.00			1.00		
**Wearing mask when social distancing is difficult**						
Bad (0 point)	1.82	(1.14–2.91)	0.012	1.44	(0.93–2.23)	0.099
Good (1 point)	1.00			1.00		

Adjusted with all covariates.

*PHQ-9* Patient Health Questionnaire-9; *aOR* adjusted odds ratio; *CI* confidence interval.

Table 4 shows the stratified analysis according to education level. In the case of the people who did not graduate high school, in both sexes, the performance score was not associated with depression. However, in the case of the people who graduated high school, the adjusted OR values of bad performance scores were largest in both sexes (Men: aOR = 2.45, 95% CI: 1.29–4.65, Women: aOR = 3.75, 95% CI: 1.73–8.13).

**Table 4:** Association between COVID-19 quarantine rules performance score and depression according to the education level.

Variables	Depression (PHQ-9 ≥ 10)					
	Men			Women		
	aOR	95% CI	*p*-value	aOR	95% CI	*p*-value
**Under high school**						
**Covid-19 quarantine rules Performance Score**						
Bad (0–1 point)	1.81	(0.68–4.82)	0.234	1.49	(0.77–2.91)	0.238
Moderate (2 points)	1.33	(0.78–2.25)	0.297	1.06	(0.81–1.38)	0.684
Good (3 points)	1.00			1.00		
**Graduated high school**						
**Covid-19 quarantine rules Performance Score**						
Bad (0–1 point)	2.45	(1.29–4.65)	< 0.001	3.75	(1.73–8.13)	0.001
Moderate (2 points)	1.29	(0.97–1.71)	0.079	1.36	(1.09–1.71)	0.007
Good (3 points)	1.00			1.00		

Adjusted with all covariates.

*PHQ-9* Patient Health Questionnaire-9; *aOR* adjusted odds ratio; *CI* confidence interval.

## Discussion

Depression is a leading cause of disability worldwide, and the prevalence of depression in countries around the world has doubled since 2020^18,32^. Furthermore, the prevalence of depressive symptoms (PHQ-9 score ≥ 10) in South Korea after COVID-19 pandemic (18.8%) is significantly higher than the rates of 6.1–6.7% reported in previous Korean studies that analyzed population-based data^33^. The increase in the prevalence of depressive symptoms in Korea is larger than the 9.1% increase reported in a US study^34^. Therefore, it is important to investigate factors related to depressive symptoms in Korea after commencement of the COVID-19 pandemic.

Under these circumstances, the present study investigated the association of compliance with COVID-19 public health measures with depression using PHQ-9. Our findings indicated that there was a significant association between compliance as measured by a performance score and depression. In other words, compared to those who completely followed the quarantine rules, those who did not follow even one were more likely to be depressed.

Several previous articles and studies have demonstrated that the number of people who have depression has increased worldwide due to COVID-19^35^. Some studies have explained that depression is caused due to social isolation, lower income, or fears of infection^36–38^. Direct biological effects from coronavirus have also likely contributed to the increased prevalence of depression during the COVID-19 pandemic. Previous studies have reported that coronavirus can directly penetrate the central nervous system or leave psychopathological sequelae through the immune system^39,40^. However, few studies have investigated the relationship between COVID-19 and depression by focusing on the compliance of quarantine measures that have become a daily routine because of COVID-19.

Several possible theories support our results. First, anxiety about disease transmission from not following quarantine guidelines can lead to depression. People who perceive themselves to be at higher risk of exposure to the virus are more likely to report symptoms indicative of depression^41,42^. In a subgroup analysis, the association between wearing a mask indoors and depression had a higher odds ratio in association with depression than not wearing a mask outdoors and not practicing social distancing. It is well known that wearing a mask can reduce the transmission of COVID-19^43,44^, and that the virus spreads better indoors than outdoors^45^. People who re-used masks had stronger beliefs about the severity of the COVID-19 disease and were more likely to experience depressive symptoms. In addition, a recent study reported that students who did not wear masks had greater psychological stress compared to those who wore masks^46^. Considering these points, people who do not wear a mask indoors are more likely to have depressive symptoms because of fear that they may contract an infectious disease even if they choose not to wear a mask.

Second, compliance with quarantine rules can provide an environment that is a little freer from the stress of COVID-19 pandemic. In other words, compliance with quarantine rules may be related to a decrease in the prevalence of depressive symptoms by reducing neuroinflammation possibly induced by stress^47^. Another possibility is that people's state of mind can also affect their mental health, such as depression, when making rules-following decisions. In other word, those people who followed the COVID-19 quarantine rules were happy, but those who did not follow the rules can become anxious, which can affect their mental health^48^.

Furthermore, the relationship between rule-following and mental health differed by education level. In the stratified analysis, bad COVID-19 quarantine rules performance score was significantly associated with depression among participants with higher education levels. However, there was no association between non-compliance with quarantine rules and depression in participants with lower education level. A possible explanation for these results is that education level affects hygiene practices and the will to follow the rules. This aspect requires further investigation.

There are several limitations to be considered in our study. First, owing to the cross-sectional design of the study, we cannot be confident that the PHQ-9 data collected specifically measure COVID-19-related depressive symptoms. This is because it is impossible to differentiate between pre-existing depressive symptoms and those recently caused by COVID-19. Second, as people may not have answered the survey honestly, nonrandom misclassification may have been produced^31^. This may have been the case because adherence to rules is a sensitive issue. One study showed that respondents sometimes lie in questionnaires, especially when a question is socially sensitive^49^. We were not able to adjust for this possibility in our study. Finally, the study’s cross-sectional nature did not allow us to clearly identify the direction of the relationship between compliance with COVID-19 public health measures and depression. Further longitudinal studies are required to establish a causal relationship. However, our results can be used as a basis for other related studies because our study used a methodology suitable for the dataset and adjusted for covariates associated with quarantine rule compliance and depressive symptoms.

Despite these limitations, this study has strengths. Our findings may be socially important. COVID-19 public health measures are currently major issues worldwide and it is clear that the prolonged COVID-19 pandemic has adverse effects on mental health^50,51^. Now is the time to study the effect of quarantine rules that we have to adapt to due to COVID-19 on mental health, and our research is at the starting line. Overall, non-compliance with quarantine rules was associated with depression, and this association was stronger with higher education levels. Further research on the mechanism by which the observance of quarantine rules helps mental health is necessary, and it is necessary to communicate and publicize information that observing quarantine rules can protect mental health as well as infection from COVID-19.

## Conclusions

Men and women who do not comply with public health measures during COVID-19 pandemic are likely to be depressed. Furthermore, not wearing a mask indoors showed the highest association with depression. The association between non-compliance with quarantine rules and depression was more pronounced in participants with a high level of education. These results suggest that compliance with COVID-19 quarantine rules can help mental health. Therefore, it is necessary to make it known that the development of evidence-based quarantine rules that can reduce the transmission of COVID-19 and adherence to them can be beneficial to physical and mental health.

## Data Availability

The dataset used in this study is publicly available on the CHS’s official website (https://chs.kdca.go.kr/chs/).
